# Lean Behavioral Health: The Kings County Hospital Story

**DOI:** 10.1192/pb.bp.114.048405

**Published:** 2016-02

**Authors:** Sarah Cornick

With ever increasing pressure on budgets and an emphasis on quality, safety and efficiency of healthcare services, quality improvement has become part of everyday practice in the UK, making this book a particularly topical read. Lean is a way of thinking derived from the Toyota car industry, which looks to define value from a customer's perspective and create value by eliminating waste throughout the chain of processes required to deliver a product. The idea of continuous improvement is inherent within a lean system. Lean systems have been utilised in the manufacturing sector with varying degrees of success, but more recently the principles and techniques of lean have been transferred to other sectors, including healthcare. This book outlines how lean principles and tools were utilised to lift psychiatric services at The Kings County Hospital out of crisis and promote a culture of continuous improvement in the quality of services delivered.

The book was not always the easiest read and there were several sections where a further copy-edit would have been beneficial. It took time to become familiar with the language of lean and the acronyms used, although a glossary at the start of the book assisted in the learning process. Earlier chapters set the scene, explaining lean and the background to the crisis facing the hospital, and gave brief details of many improvement projects that were undertaken. However, at this point the exact nature of problems within different departments, solutions identified and how lean methodologies had assisted the process were not always clear. Later chapters revisited some of the examples on a department-by-department basis and gave a better sense of the value of lean tools in identifying and solving problems with the aim of sustainable change and creation of a culture of continuous improvement. Figures supplementing the text gave an indication of the scope of various lean events but it was impossible to read the detail of what had been discussed due to their small size. Although this book was not aiming to act as a manual for the application of lean in a healthcare setting, at times it was frustrating that detail in examples and figures was lacking because this would have strengthened understanding of the difficulties facing the organisation prior to and during change events and the value of lean in helping to identify solutions and guide teams through the change process.

At The Kings County Hospital, application of lean thinking typically involved initial work to establish the current state of all activities in a process requiring improvement, creation of a target state, and corresponding gap analysis between the two states. Identified problems were then addressed through rapid improvement events (during the course of a 4.5-day event waste was identified, solutions tested and improvements made), longer projects and immediate ‘just do its’. Evidence was provided that where lean principles were successfully implemented within the organisation, there were consequent benefits: financially, to staff productivity and morale, to the quality, safety and efficiency of clinical services delivered, and in the experience of patients. For example, in the psychiatric emergency room, the physical flow of patients through the department and collection of patient data were streamlined, with clear standards of work developed for staff involved in each step in the process. This led to elimination of previous duplication of work, shorter nursing and psychiatric triage times and improved standards of patient care.

Overall, the book did provide a thought-provoking and, at times, inspirational commentary on real-life and wide-ranging changes to working practices across the hospital's psychiatric emergency, in-patient and out-patient services. It demonstrated how improvement methodologies can help to establish a new organisational culture, reduce waste and improve quality in any process pathway. As well as commenting on successful changes, the book also offered insights into changes which had not worked so well and reasons for that, acknowledging that the process of improvement is ongoing and that the organisation continues to strive towards an ideal state. The book offers a good case study as a starting point for anyone interested in incorporating improvement methodologies at any level of an organisation, demonstrating the benefits of thinking outside of the healthcare box and utilising successful quality and value improvement strategies developed in other sectors.

